# Letter to the Editor: “Detection of Ribosomal DNA Sequence Polymorphisms in the Protist *Plasmodiophora brassicae* for the Identification of Geographical Isolates”

**DOI:** 10.3390/ijms18071454

**Published:** 2017-07-06

**Authors:** Arne Schwelm, Sigrid Neuhauser

**Affiliations:** 1 Department of Plant Biology, Uppsala BioCenter, Linnean Centre for Plant Biology, Swedish University of Agricultural Sciences, P.O. Box 7080, SE-75007 Uppsala, Sweden; 2 Institute of Microbiology, University of Innsbruck, Technikerstraße 25, 6020 Innsbruck, Austria; sigrid.neuhauser@uibk.ac.at

To the editor,

In the publication “Detection of Ribosomal DNA Sequence Polymorphisms in the Protist *Plasmodiophora brassicae* for the Identification of Geographical Isolates”, Laila et al. investigated the ribosomal sequences of field isolates from *Plasmodiophora brassicae* [1]. The authors report a putative polymorphism in the large ribosomal subunit (LSU) in Korean isolates (800–2800 bp into the LSU). The authors conducted their research based on the finding reported in 2011 by Niwa et al. [2], in which they reported polymorphism in DNA extracted from Japanese *P. brassicae* infested field/soil samples. However, recently we showed in [3] that the polymorphism described in [2] was due to chimeric PCR products starting from 850 bp into the LSU. We confirmed the lack of polymorphism of several *P. brassicae* isolates in this region from all over the world (including Korea) which was backed up by the genomic sequence of *P. brassicae* [4] and fluoresence in situ hybridisation (FISH) experiments [3]. The LSU polymorphism reported by Niwa et al. [2] is a result of a chimeric PCR product which in 2011 was not identifiable based on sequences that were publicly available. New and better taxon sampling of LSU sequences of Rhizaria combined with more phytomyxea sequences identified that ca. 75% of the LSU sequence (GenBank Accession Numbers AB526843) reported by [2] originated from another Cercozoan, most likely a Glissomonadida [3]. Due to this issue, the authors of [2] have removed AB526843 from Genbank.

As Laila et al. designed their primers on the accession AB526843 they produced similar chimeric products. Laila et al. report that “No sequence variation was observed among 11 Korean field isolates and published sequences for the first 870 sequences of LSU (Figure S4), i.e., including SSU, ITS and 5.8 s about 4425 bp rDNA sequences of 11 Korean field isolates were identical with three published sequences AB526843, KX011115 and KX011135”. This is in accordance with the above-mentioned polymorphism, that starts at 4422 bp or 850 bp downstream the LSU. We build a phylogentic tree as in [3] with the same Cercozoan sequences and with the alignment reduced to the area where all the sequences overlap to avoid effects caused by the non-chimeric identical part (Figure 1). This shows that the 1.4 kb from the sequences by [1] are also not of *P. brassicae* or Phytomyxea origin, but very likely belong to free living soil flagellates instead (as in case of the AB526843 likely of Glissomonadida origin).

However, the high resolution melting (HRM) results reported by Laila et al. [1] are probably not influenced by this as only the last three probes listed in Table 3 are in the chimeric part of the sequence, but this would explain that the HRM experiments did not give conclusive results with primers designed to the upstream part of the sequences. We would like the authors to carefully recheck and revalidate the sequences obtained, using new designed primers, which are based on the *P. brassicae* LSU sequences (KX011115-KX011135) or the genome sequences of *P. brassicae* available to date. This would provide other researchers with valuable resources on the ribosomal sequences in the Korean *P. brassicae* populations. *P. brassicae* is an economically important pathogen and many researchers work on detection and identification methods using ribosomal sequences. We think it is important to correct the sequences in the public databases. This would prevent researchers from designing their studies based on misleading data and designing specific markers that then will detect very common soil flagellates rather than this important plant pathogen.

## Figures and Tables

**Figure 1:**
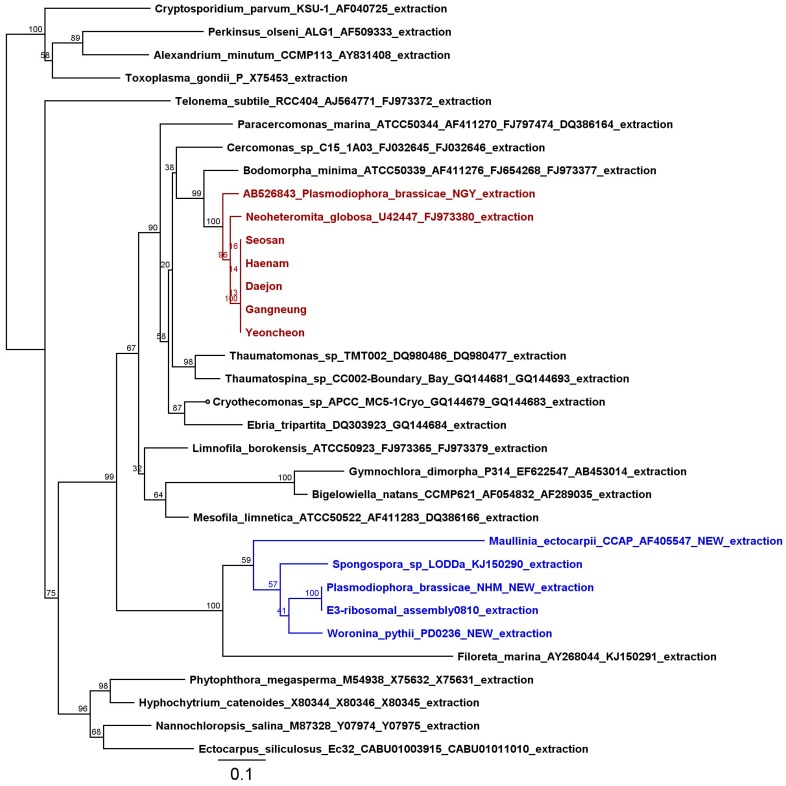
Phylogeny of the ribosomal DNA sequences of Cercozoan and the last 1.4 kb of the sequences reported by Laila et al. [1] aligned to the reference set as in [3]. Shown is a RAxML analysis using GTR Gamma I Nucleotide Model and rapid bootstraping with search for best scoring Maximum Likelihood phylogenetic tree, starting from a complete random tree. Node display values of 100 bootstraps. Phytomyxea/Plasmodiophorida are marked in blue and the chimeric sequences reported by [1], the according part of AB526843 and a Glissomonadida are highlighted in red. Our tree was generated using only the part of the 3′-end LSU we believe is chimeric. This avoids any distorted branching pattern, which would undoubtedly be the case using the full length rDNA gene.
